# Factors related to intrapartum/delivery care in Southeast Asia: A cross-sectional study in the Philippines and Indonesia

**DOI:** 10.1016/j.heliyon.2024.e27718

**Published:** 2024-03-08

**Authors:** Ratna Dwi Wulandari, Agung Dwi Laksono, Nikmatur Rohmah, Ratu Matahari, Carl Abelardo Antonio

**Affiliations:** a Universitas Airlangga, Surabaya, Indonesia; b The Airlangga Centre for Health Policy (ACeHAP) Research Group, Surabaya, Indonesia; c National Research and Innovation Agency Republic of Indonesia, Jakarta, Indonesia; d Universitas Muhammadiyah Jember, Jember, Indonesia; e Universitas Ahmad Dahlan, Yogyakarta, Indonesia; f University of the Philippines Manila, Manila, Philippines

**Keywords:** Intrapartum/delivery care, Maternal health, Women's health, Southeast Asia, Public health

## Abstract

**Background:**

Policy encouraging healthcare intrapartum/delivery care is critical to accelerating the decline in maternal mortality. The study analyzes intrapartum/delivery care factors in Indonesia and the Philippines.

**Methods:**

The investigation included 15,346 Indonesian and 7992 Filipino women (ages 15 to 49 who delivered during the previous five years). Aside from the location of intrapartum/delivery care as a dependent variable, additional factors investigated included domicile, marital status, age, occupation, education, parity, wealth, and ANC—the conclusion of the study utilizing binary logistic regression.

**Results:**

Women in both countries predominantly do healthcare intrapartum/delivery care. Both countries' urban women are more likely to receive intrapartum/delivery care than rural women. The higher the amount of schooling, the greater the likelihood of receiving intrapartum/delivery care. The lower the parity, the higher the chance to do healthcare intrapartum/delivery care. The higher the wealth position, the greater the likelihood of receiving intrapartum/delivery care. Furthermore, women in both nations who had four or more antenatal visits were more likely to receive intrapartum/delivery care.

**Conclusion:**

The study concluded five factors related to healthcare intrapartum/delivery care in the Philippines: residence, education, parity, wealth, and ANC. Meanwhile, there are six factors related to healthcare intrapartum/delivery care in Indonesia: place, age, education, parity, wealth, and ANC.

## Highlights

• The study analyzes factors related to delivery care in Indonesia and the Philippines.
• Women in two countries predominantly do healthcare delivery care.
• Related factors in the Philippines are place, education, parity, wealth, and ANC.
• Related factors in Indonesia are place, age, education, parity, wealth, and ANC.

## 1 Introduction

A maternal death implies a mortality caused by problems during pregnancy or delivery. The maternal mortality rate (MMR) in Indonesia is 177 deaths per 100,000 live births, making it the region's third largest in Southeast Asia behind Myanmar and Laos. The Philippines has Southeast Asia's sixth-highest MMR (121 deaths per 100,000 live births). Meanwhile, Vietnam, Thailand, Brunei, Malaysia, and Singapore are the five other Southeast Asian countries with an MMR below 100 per 100,000 live births [1].

From 2000 to 2017, MMR globally decreased by 38%, meaning the average yearly decline was 2.9%. The target for reducing MMR per year is 6.4%, needed to achieve the global sustainable development goals (SDGs) of 70 maternal deaths per 100,000 live births. From 2010 to 2017, MMR in Indonesia declined by 7–8 fatalities per 100,000 newborns, or 3%. The MMR in the Philippines also experienced a gradual decline from 2003 to 2017. The MMR in the Philippines peaked at 156 fatalities per 100,000 live births in 2003 and in 2017 fell to 121 deaths per 100,000 live births. The falling trend in MMR in the Philippines and Indonesia is still not meeting the SDGs objective by 2030 [2,3].

Several studies identified several causes of the high maternal mortality rate as direct and indirect causes. Studies in various countries have also been conducted on the causes of MMR. Cameroon studies show maternal mortality declines with age and higher education [4]. Meanwhile, the high MMR in Indonesia is caused by barriers to access and features of health services, which represent 23% of the overall population [5]. Furthermore, economic class, caste/ethnicity, education, religion, gender, and culture are the most significant structural determinants influencing MMR in India. Other causes include the location of living, maternal age at delivery, parity, exposure to advice from the media, and the health establishment [6]. MMR's direct causes in India include preeclampsia and eclampsia, severe anemia, hepatic encephalopathy, septic abortion, postpartum hemorrhage, complications during childbirth, and the puerperium [[7], [8], [9]]. Other causes of maternal mortality include living in slums, primiparous, assisted delivery by traditional birth attendants, preference for home delivery, shame in repeated pregnancies, fear of prejudice from providers of services, history of effective home delivery, overconfidence, and not accessing care in healthcare facilities [8,10]. Another study in Indonesia stated that deliveries outside a health facility ranged from 21 to 60% [11]. The research shows that health facilities still have obstacles to the delivery target.

The policy to reduce MMR aims to minimize direct and indirect causes. Some recommendations related to intrapartum/delivery care policies include increasing delivery in health facilities, increasing women's understanding of the positive aspects of giving childbirth in a health facility, as well as encouraging men to use maternal health care. Furthermore, enhancing women's decision-making options reduces barriers caused by the absence of availability of transportation, enhances accessibility to healthcare resources, and improves health education by concentrating on the hazards of threatening birth at home [9,12]. The challenge in setting intrapartum/delivery care policies is the accessibility and affordability of delivery services in health facilities, which are culturally acceptable [10]. Providing adequate delivery infrastructure should also follow policies encouraging care delivery in health facilities.

The challenge in reducing barriers to midwifery care is not only related to the accessibility of childbirth services in healthcare centers. A poll conducted in the Kampala urban area of Uganda stated that although physical access was good, 33.9% of mothers did not deliver birth in a medical facility [13]. The condition refers to an immediate requirement to boost childbirth at medical centers by assuring mandatory schooling for all women, promoting more visits to the ANC, and increasing medical center visits via networking sites and ongoing media outreach efforts [10,14]. The governments can improve a policy through interventions to raise awareness, initiate ANC early in pregnancy, and attend at least four ANC visits. Thus, we can carry out comprehensive midwifery care services systematically and sustainably in collaboration with various related parties. Until now, no study has compared the factors related to intrapartum/delivery care between the Philippines and Indonesia. This study is essential for policies to reduce maternal mortality in both the Philippines and Indonesia. Regarding context narration, the investigation will analyze the factors related to intrapartum/delivery care in Indonesia and the Philippines.

## 2 Materials and methods

### 2.1 Data Source and study design

This was a cross-sectional investigation. The author based the analysis on secondary data from the 2017 Indonesian Demographic Health Survey (IDHS) and the 2017 Philippine National Demographic Health Survey (PNDHS). The polls were a component of the Inner City Fund's international Demographic and Health Survey (DHS) program. Furthermore, the investigation employs stratification and multistage random sampling to acquire samples.

The Master Sample Frame (MSF), developed and produced by the Philippine Statistics Authority (PSA), served as the sample frame for the 2017 PNDHS. The Philippines is classified into 17 administrative regions by the study, which further subdivides the country into provinces, highly urbanized cities (HUC), and other distinct areas. There are 33 HUCs, 81 sections, and three more special zones in the Philippines. In the Philippines, there are 42,036 barangays, of which 5697 are urban and the remainder rural. The 2010 Census of Population and Housing (CPH) data were used to generate the MSF's major sample units (PSUs), which were revised in August 2015 utilizing data from the 2015 Census of Population. The survey employed the 2015 Enumeration Areas Reference File (EARF) to reassemble the PSUs, whereas the Secondary Sampling Unit (SSU) frame was derived from the 2015 dwelling unit lists. A PSU can consist of two or more nearby small barangays, a barangay, or a portion of a larger barangay. 42,036 barangays are home to about 87,098 PSUs (the survey identified 910 barangays as the least accessible; these barangays were not included in the MSF) [15].

To obtain statistics that accurately reflect the women's distribution in the Philippines, the sample must be mathematically modified, or weighted, to approximate the real distribution within the nation. The contribution of women from small regions, such as the MIMAROPA Region, to the national total should be negligible. Large-area women like those in the National Capital Region ought to make far larger contributions. Therefore, to ensure that each region's contribution to the total is proportionate to the actual population of the area, DHS statisticians statistically compute a “weight,” which is used to alter the number of women from each region [15].

However, the 2017 IDHS employed the following stratified two-stage sampling design: Step 1: based on the size of the households listed in the 2010 population census, some census blocks are selected in a methodically proportionate to size likelihood; In this instance, census blocks were sorted according to the wealth index category of the 2010 population census data, and an implicit stratification method based on urban and rural regions was used. Based on updated census block households, Stage 2 chooses 25 typical households in each census block [16].

To generate data typical of Indonesia, the distribution of women in the sample has to be weighted (or statistically adjusted) to make it reflect the proper distribution in the nation. Only a modest portion of the national total should come from women from small regions like North Kalimantan. Large-area women ought to give substantially more. Because of this, the statisticians at DHS calculate a “weight,” which is then utilized to modify the number of women from each province such that each province's share of the total is commensurate with its real population [16].

Women who had given birth in the last five years, ages 15 to 49, made up the analytic unit for the present research. The study obtained 7992 Philippines and 15,346 respondents in Indonesia by unit analysis criteria.

### 2.2 Dependent variables

Intrapartum/delivery care was a dependent factor in the research investigation. There are two categories of locations for intrapartum/delivery care: non-healthcare settings and healthcare centers. Healthcare facilities that provide intrapartum/delivery care comprise hospitals, clinics, maternity hospitals, and healthcare centers (Puskesmas) [17].

### 2.3 Independent variables

Meanwhile, the study employed seven independent variables. The seven were the residence, marital status, age group, employment status, education level, parity, wealth status, and antenatal care. The residence type consists of rural and urban categories, and the categorization refers to the Philippine Statistics Authority and Statistics Indonesia [15,16].

By recognizing the respondent's most recent credentials, the study was able to ascertain their degree of education, which falls into four categories: primary, secondary, higher, and no education. Age is determined by the respondent's most recent birthday. Seven groups, spanning five years, make up the age group: 15–19, 20–24, 25–29, 30–34, 35–39, 40–45, and 45–49 [14]. There are three types of marital status: never married, married and cohabiting, and divorced or widowed. The number of children born and alive was known as parity. There are three types of parity: grand multiparous (>4), multiparous (2–4), and primiparous (<1) [18].

The wealth quintile that belonged to families was used by the survey (the 2017 PNDHS and the 2017 IDHS) to establish wealth status. Based on the quantity and kind of items people owned, the survey assigned a belongings grade. It covers everything from televisions to vehicles, bikes, or bicycles, as well as residential features like restrooms, drinking water supplies, and the main components of the floor. In the survey, principal component analysis was used to determine the score. Based on household scores for each household, the survey produced national wealth categories, which were subsequently distributed into the same five sections and represented 20% of the entire population. The most impoverished, less wealthy, middle, more prosperous, and most prosperous are the five categories of wealth [19,20].

Despite 2016 WHO guidelines changing the recommended minimum number of ANC contacts from four to eight, including four ANC visits between 8 and 12 weeks of gestation, between 24 and 26 weeks, at 32 weeks, and between 36 and 38 weeks, the governments of Indonesia and the Philippines still use the basic ANC approach [21]. This study divides the ANC into two groups: <4 ANC visits and ≥4 ANC visits, according to these regulations.

### 2.4 Data analysis

Chi-square analysis was used in the early phases to examine various variables and features related to intrapartum/delivery care. Because of the nature of the dependent variable, the study's last stage was using binary logistic regression to calculate the odds ratio with a 95% confidence interval (CI). For all phases of the statistical review, SPSS IBM Statistics 26 software was utilized.

### 2.5 Ethical approval and consent to participate

For the materials analysis, the investigators used secondary data from the 2017 IDHS and PNDHS. Every respondent's identity is eliminated from the dataset by the surveys. We adhere to all applicable rules and regulations while using any method. All participants and their legal guardians provided written informed consent for the questionnaires in the interim. The author obtained authorization to use data from the https://dhsprogram.com website for this study.

For the 2017 PNDHS and the 2017 IDHS, all testing procedures follow the Standard DHS survey technique under The Demographic and Health Surveys (DHS) Program (DHS-7), which is approved by the ORC Macro Institutional Review Board 2002 after being first assessed and approved by the IRB of ICF International. The IRB adhered to the 45 CFR 46 guidelines for “Protection of Human Subjects” established by the US Department of Health and Human Services. DHS investigations that comply with the standards are categorized as DHS-7 Program authorized, along with the necessary certification documentation.

## 3 Results

The analysis found that women in the Philippines who did intrapartum/delivery care at healthcare facilities are 77.5%. Meanwhile, pregnant women in Indonesia perform intrapartum/delivery care at healthcare facilities at 75.1%.

Table 1 presents the crosstabulation outcome between intrapartum/delivery care and other factors. According to residence type, Philippine women who do intrapartum/delivery care in facility care predominantly live in rural areas. In contrast, Indonesian women who do intrapartum/delivery care in the facility tend to live mostly in urban regions.

**Table 1:** The crosstabulation outcome between intrapartum/delivery care and other variables in Indonesia (n = 15,346) and the Philippines (n = 7992).

Variables	Philippine (n = 7992)			Indonesia (n = 15,346)		
	Non Healthcare Facilities (1,796)	Healthcare Facilities (6,196)	p-value	Non Healthcare Facilities (3,831)	Healthcare Facilities (11,526)	p-value
Type of residence			**<0.001			**<0.001
• Urban	20.3%	36.1%		23.9%	57.7%	
• Rural	79.7%	63.9%		76.1%	42.3%	
Age group			**<0.001			**<0.001
• 15 - 19	3.8%	4.4%		3.7%	2.4%	
• 20 - 24	19.5%	20.4%		16.9%	15.3%	
• 25 - 29	26.4%	26.4%		24.1%	25.4%	
• 30 - 34	19.2%	21.9%		24.8%	26.2%	
• 35 - 39	16.9%	16.5%		18.9%	20.3%	
• 40 - 44	10.5%	8.3%		9.0%	8.8%	
• 45 - 49	3.6%	2.1%		2.6%	1.7%	
Marital status			**<0.001			*0.018
• Never in union	1.7%	4.5%		0.3%	0.2%	
• Married/living with a partner	95.0%	91.8%		96.0%	96.9%	
• Divorced/Widowed	3.3%	3.7%		3.7%	2.9%	
• Education Level			**<0.001			**<0.001
• No education	4.7%	0.5%		3.4%	0.7%	
• Primary	38.9%	13.3%		39.6%	20.3%	
• Secondary	44.4%	50.8%		47.8%	59.0%	
• Higher	12.0%	35.4%		9.2%	20.0%	
Employment status			**<0.001			*0.038
• Unemployed	63.6%	58.5%		53.9%	52.0%	
• Employed	36.4%	41.5%		46.1%	48.0%	
Parity			**<0.001			**<0.001
• Primiparous	16.4%	30.7%		24.4%	33.2%	
• Multiparous	53.0%	56.1%		60.8%	61.5%	
• Grand multiparous	30.6%	13.2%		14.8%	5.4%	
Wealth status			**<0.001			**<0.001
• Poorest	60.3%	25.2%		52.5%	17.9%	
• Poorer	22.3%	24.5%		20.8%	19.4%	
• Middle	11.1%	20.1%		13.6%	20.6%	
• Richer	4.6%	17.1%		9.1%	20.9%	
• Richest	1.7%	13.2%		3.9%	21.2%	
Antenatal care			**<0.001			**<0.001
• <4 visits	36.3%	9.9%		25.2%	6.7%	
• ≥4 visits	63.7%	90.1%		74.8%	93.3%	

Note: *p < 0.050; **p < 0.001.

Considering the age range, the 25–29 ruled Philippine women who performed intrapartum/delivery care in facility care. Meanwhile, the 30–34 dominant Indonesian women perform intrapartum/delivery care in facility care. Concerning marital status, married/living with partner women are prevalent in both countries, serving intrapartum/delivery care in facility care. Furthermore, based on education level, women in both countries who do intrapartum/delivery care in facility care have a dominant secondary education.

According to employment status, unemployed women in both countries perform intrapartum/delivery care in facility care. Both countries ruled multiparous women performing intrapartum/delivery care in facility care based on parity.

Philippine women perform intrapartum/delivery care in facilities populated by the most impoverished women according to their financial standing. Meanwhile, more prosperous women settled in Indonesian women who perform intrapartum/delivery care in facility care. Women who did intrapartum/delivery care in facility care were occupied by women who made ANC visits four times regarding antenatal care.

Table 2 provides the binary logistic regression results of intrapartum/delivery care in Indonesia and the Philippines. Based on the type of residence, the result shows that both countries have the same tendency. Women who reside in cities are likelier to use hospitals for intrapartum/delivery care.

**Table 2:** The binary logistic regression outcome of intrapartum/delivery care in the Philippines (n = 7992) and Indonesia (n = 15,346).

Variables	Healthcare Facilities					
	Philippine			Indonesia		
	Adjusted Odds Ratio	95% Confidence Interval		Adjusted Odds Ratio	95% Confidence Interval	
		Lower Bound	Upper Bound		Lower Bound	Upper Bound
Type of residence						
• Urban	**1.221	1.054	1.414	***2.359	2.146	2.594
• Rural (ref.)	–	–	–	–	–	–
Age group						
• 15–19	1.190	0.733	1.932	**0.577	0.392	0.850
• 20–24	0.945	0.639	1.399	*0.705	0.512	0.972
• 25–29	0.932	0.640	1.356	0.824	0.606	1.119
• 30–34	1.145	0.790	1.660	0.946	0.699	1.280
• 35–39	1.121	0.775	1.622	1.130	0.836	1.527
• 40–44	1.222	0.830	1.799	1.279	0.933	1.754
• 45–49 (ref.)	–	–	–	–	–	–
Marital status						
• Never in union (ref.)	–	–	–	–	–	–
• Married/living with a partner	0.674	0.448	1.015	0.682	0.295	1.576
• Divorced/Widowed	0.619	0.370	1.035	0.635	0.268	1.506
Education Level						
• No education (ref.)	–	–	–	–	–	–
• Primary	**1.911	1.223	2.987	*1.387	1.004	1.917
• Secondary	***3.767	2.413	5.882	***1.963	1.420	2.714
• Higher	***5.351	3.347	8.555	***2.043	1.446	2.886
Employment status						
• Unemployed (ref.)	–	–	–	–	–	–
• Employed	0.989	0.871	1.124	0.955	0.876	1.041
Parity						
• Primiparous	***2.143	1.685	2.726	***2.972	2.460	3.591
• Multiparous	***1.487	1.249	1.771	***1.839	1.577	2.145
• Grand multiparous (ref.)	–	–	–	–	–	–
Wealth status						
• Poorest (ref.)	–	–	–	–	–	–
• Poorer	***1.815	1.567	2.103	***1.932	1.733	2.154
• Middle	***2.396	1.984	2.892	***2.529	2.237	2.860
• Richer	***3.917	3.014	5.089	***3.145	2.727	3.628
• Richest	***6.881	4.615	10.260	***6.104	5.017	7.426
Antenatal care						
• < 4 visits (ref.)	–	–	–	–	–	–
• ≥ 4 visits	***3.486	3.029	4.012	***3.057	2.724	3.430

Note: *p < 0.050; **p < 0.010; ***p < 0.001.

Regarding the age group, the analysis of the results indicated non-significant results in the Philippines. Meanwhile, partially significant results have been shown in Indonesia with intrapartum/delivery care. Women in the 15–19 and 20–24 age groups had a lower probability of performing intrapartum/delivery care at a healthcare facility than women aged 45–49. According to marital status, the analysis results in both countries indicate an insignificant relationship with intrapartum/delivery care.

The analysis results found consistent trends in the two countries regarding education level. Higher education levels increase the likelihood of intrapartum/delivery care at a healthcare facility. According to employment status, the analysis results in both countries indicate an insignificant relationship with intrapartum/delivery care.

The study results indicated the same trend in both countries based on parity. The likelihood of intrapartum/delivery care at a healthcare facility increases with decreasing parity. In line with the education level, the analysis results on wealth status also found the same trend in the Philippines and Indonesia; better financial standing increases the likelihood of intrapartum/delivery care at healthcare facilities.

Moreover, the two countries show a similar pattern regarding ANC contacts. Women were more likely to receive intrapartum/delivery care at an institution if they had four or more ANC appointments.

## 4 Discussion

In response to maternal health challenges, the Philippine government institutionalized a national policy (The 2012 Responsible Parenthood and Reproductive Health Act or Republic Act 10354) that, among others, advanced the welfare of women throughout their pregnancies, deliveries, and postpartum phases. This policy's implementation strategy establishes service delivery networks linking different levels of care that a woman and her child may need throughout pregnancy, childbirth, and postpartum [22,23]. This strategy, however, is hampered by resource constraints, the country's geopolitical landscape, and a devolved health system [24].

Meanwhile, to lower Indonesia's MMR, the Health Ministry has established five operational strategies, namely strengthening Puskesmas and their networks; strengthening program management and its referral system; increasing community participation; cooperation and partnership; acceleration and innovation activities; collaborative innovation research and development [25,26]. The government also set new standards for good delivery, assisted by health workers to deliver in health facilities [27,28].

The study found that women in both countries dominated intrapartum/delivery care at healthcare facilities. This situation means that women in Indonesia and the Philippines are highly aware of doing ANC and giving birth in maternal health services. The findings of this investigation concur with a maternal health study in Thailand, which showed a shift in Thai women's understanding and mindset regarding access to maternal health services. The situation indicates that Thai women are highly aware of checking and giving birth in healthcare facilities [29]. The hospital is a maternal health service women trust to be safe and comfortable because it has a complete health infrastructure, and professional health workers support the excellent services [30,31].

According to the study's findings, women in both nations who reside in metropolitan areas are more likely to receive intrapartum and delivery care at medical institutions. Due to this condition, women who live in neighborhoods have more access to maternal health services than women who live in rural regions, who have less access to such services [28,[32], [33], [34]]. Meanwhile, studies in Nigeria and Indonesia show those ladies who reside in rural regions influence local beliefs related to maternal care, so they prefer to perform ANC on traditional attendants [35,36]. On the other hand, the findings also conform with the study in Ethiopia. The study found that some factors, such as limited access to transportation and a shortage of qualified healthcare providers, contribute to rural women's reluctance to seek maternity care in health facilities [37]. However, the outcomes differ from studies conducted in Nigeria. The study showed that Nigerian women who reside in remote locations tend to give birth to traditional birth attendants to health workers in health services. The reasons include the high cost of maternal health services, medicine affordability, and health professionals' poor attitude compared to traditional birth attendants [38].

The results revealed that the likelihood of receiving intrapartum/delivery care at a hospital increased with education level. This finding is different from previous studies in which women with high and low levels of education have the awareness to perform ANC and give birth in maternal health services [29,39]. In addition, the husband's education level also plays an essential role in conducting ANC examinations in health services. The husband's education level at the senior high school level will support couples in checking ANC in health services compared to primary school education. The condition applies to the husband's higher education to keep the couple doing ANC checks in health services [40,41]. Education level is also related to a person's perception of the quality of healthcare centers [42,43]. Several investigations have indicated that increased educational attainment is a significant predictor of improved health outcomes [[44], [45], [46]]. Conversely, a lack of education stands in the way of achieving improved health outcomes [47,48].

The study results indicated the same trend in both countries based on parity. The likelihood of intrapartum/delivery care at a medical facility increases with decreasing parity. The findings are consistent with earlier research that shows the smaller the number of parity, the more people will have to check their pregnancy in health services [49,50]. A survey in Ethiopia also showed that women's parity of 1–2 who intend not to have any more children would do intrapartum/delivery care in health services and get family planning services to prevent pregnancy [37].

In the meantime, outcomes show that the likelihood of receiving intrapartum/delivery care in the hospital increases with wealth level. This condition means that someone with a better level of wealth has an increased opportunity for intrapartum/delivery care in health facilities. The findings are consistent with a study conducted in Nigeria, which indicated that ANC in health services is performed by urban-dwelling, highly economically mobile women [51]. A person with a better level of wealth has a more significant opportunity in financing to access health services [52,53].

Finally, based on ANC visits, women in both nations were more likely to receive intrapartum/delivery care at a medical facility if they had four or more ANC visits. The situation means that complete ANC visits are associated with women's awareness of intrapartum/delivery care in healthcare facilities. Several studies have shown that age is related to incomplete ANC visits [27,54,55]. In addition, ANC visits to Indonesian women are also closely associated with family support (parents/in-laws). This situation relates to the decision-making process, which often involves an extended family [35,56].

### 4.1 Strength and limitation

The utilization of big data to portray national information in Indonesia and the Philippines is this study's strongest point. On the contrary, this study has several limitations because of the use of secondary data. Due to the lack of one or both datasets, the study is unable to investigate other variables that have been discovered in earlier investigations. Among these factors are having insurance for health, delivery type, and being aware of the warning symptoms of pregnancy [[57], [58], [59]]. Moreover, the study does not look at cultural elements and ideas that the other researchers showed to influence intrapartum/delivery care in prior studies [60,61].

## 5 Conclusion

The study concluded five factors related to healthcare intrapartum/delivery care in the Philippines: residence, education, parity, wealth, and ANC. Meanwhile, there are six factors related to healthcare intrapartum/delivery care in Indonesia: place, age, education, parity, wealth, and ANC. The author recommends that policymakers release policies focusing on policy targets following the findings of this study to accelerate intrapartum/delivery care in healthcare facilities.

Furthermore, several potential research topics can be followed up to explore other intrapartum/delivery care factors. Some are health insurance, knowledge about the pregnancy danger signs, local context, and the culture of pregnancy and childbirth.

## Funding

Not applicable.

## Data availability

Information is available from the authors upon an appropriate request and with the ICF's agreement. The 2017 IDHS and PNDHS data set name required by the ICF (data set of childbearing age women) is accessible via the website https://dhsprogram.com for investigators who fulfill the requirements for accessing sensitive data.

## CRediT authorship contribution statement

**Ratna Dwi Wulandari:** Writing – review & editing, Validation, Supervision, Project administration, Methodology, Formal analysis, Conceptualization. **Agung Dwi Laksono:** Writing – original draft, Visualization, Resources, Methodology, Investigation, Funding acquisition, Formal analysis, Conceptualization. **Nikmatur Rohmah:** Writing – original draft, Software, Resources, Project administration, Investigation, Formal analysis, Data curation. **Ratu Matahari:** Writing – original draft, Visualization, Software, Resources, Investigation, Formal analysis, Data curation. **Carl Abelardo Antonio:** Writing – review & editing, Validation, Supervision.

## Declaration of competing interest

The authors declare that they have no known competing financial interests or personal relationships that could have appeared to influence the work reported in this paper.

## Abbreviations

MMRL: maternal mortality rate
SDGs: sustainable development goals
ANC: antenatal care
DHS: Demographic Health Survey
PNDHS: Philippine National Demographic Health Survey
IDHS: Indonesian Demographic Health Survey
MSF: Master Sample Frame
CPH: Census of Population and Housing
PSUs: principal sample units
SSU: Secondary Sampling Unit
IRB: Institutional Review Board.
